# Species-dependent variation in sensitivity of *Microcystis* species to copper sulfate: implication in algal toxicity of copper and controls of blooms

**DOI:** 10.1038/srep40393

**Published:** 2017-01-12

**Authors:** Haiming Wu, Gaojie Wei, Xiao Tan, Lin Li, Ming Li

**Affiliations:** 1 College of Resources and Environment, Northwest A&F University, Yangling 712100, P R China; 2 College of Environment, Hohai University, Nanjing 210098, P R China; 3 Key Laboratory of Poyang Lake Wetland and Watershed Research, Ministry of Education, Jiangxi Normal University, Nanchang 330022, P R China; 4 Key Laboratory of Plant Nutrition and the Agri-environment in Northwest China, Ministry of Agriculture, P R China

## Abstract

Copper sulfate is a frequently used reagent for *Microcystis* blooms control but almost all the previous works have used *Microcystis aeruginosa* as the target organism to determine dosages. The aim of this study was to evaluate interspecific differences in the responses of various *Microcystis* species to varying Cu^2+^ concentrations (0, 0.05, 0.10, 0.25, and 0.50 mg L^−1^). The half maximal effective concentration values for *M. aeruginosa, M. wesenbergii, M. flos-aquae*, and *M. viridis* were 0.16, 0.09, 0.49, and 0.45 mg L^−1^ Cu^2+^, respectively. This showed a species-dependent variation in the sensitivity of *Microcystis* species to copper sulfate. Malonaldehyde content did not decrease with increasing superoxide dismutase content induced by increasing Cu^2+^, suggesting that superoxide dismutase failed to reduce Cu^2+^ damage in *Microcystis*. Considering the risk of microcystin release when *Microcystis* membranes are destroyed as a result of Cu^2+^ treatment and the stimulation effects of a low level of Cu^2+^ on growth in various species, our results suggest that copper sulfate treatment for *Microcystis* control could be applied before midsummer when *M. aeruginosa* and *M. viridis* are not the dominant species and actual amount of Cu^2+^ used to control *M. wesenbergii* should be much greater than 0.10 mg L^−1^.

*Microcystis* blooms are increasing in freshwater ecosystems as a result of eutrophication and global temperature increases, becoming a global environmental and ecological problem^1^,^2^. Besides the environmental consequences, such as unpleasant odor, loss of water transparency, and depletion of oxygen^3^, microcystin production is a serious problem resulting from *Microcystis* blooms that threatens public health through drinking water supply and fishing^4^.

Many methods, including biological^5^, chemical^6^, physical^7^, and nutrient restriction^8^, have been proposed and applied to blooms control by inhibiting *Microcystis* growth. However, most of these methods are impeded because they are expensive and slow to take effect, except for some chemical methods. Chemical methods can have serious negative effects on ecosystems, e.g., toxin release, persistence, and bioaccumulation^9^,^10^. Nevertheless, it is still considered both an effective emergency strategy and a last chance to control *Microcystis* blooms^11^.

Copper sulfate is a frequently-used, low-cost chemical reagent for blooms control and the copper sulfate toxicity mechanisms have been well studied^12^,^13^. Many lakes and reservoirs, e.g., Fairmont Lakes (USA)^14^, Myponga Reservoir (Australia)^15^, Ömerli Reservoir (Turkey)^16^, and a prairie lake in Canada^17^, were treated with copper sulfate. A moderate dose of copper sulfate, not toxic to humans or aquatic animals, to control bloom-forming *Microcystis* was chosen to inhibit *Microcystis* growth. Hadjoudja *et al*.^12^ reported that the 24-h-EC_50_ of *Microcystis* is 0.064 mg L^−1^ Cu^2+^. Tsai^18^ reported that the minimum Cu^2+^ concentration required to inhibit *M. aeruginosa* growth is 0.160 mg L^−1^. The 96-h-EC_50_ of *M. aeruginosa* was reported as 1.02 mg L^−1^ Cu^2+^ by Zhang *et al*.^19^. All of the above Cu^2+^ doses are considered safe according to the World Health Organization recommendation (2 mg L^−1^)^20^. Whereas, a recent study reported that a safe Cu^2+^ concentration (0.16–0.64 mg L^−1^) would lyse *Microcystis* cells and release microcystins from the cells^11^,^21^,^22^. Therefore, more care should be taken when recommending a safe Cu^2+^ dose and more information on the effects of Cu^2+^ on growth, physiology, and cell integrity of both toxic and non-toxic *Microcystis* species is required.

It is noteworthy that almost all of the previous studies used *M. aeruginosa* as the target organism to determine the Cu^2+^ dose for *Microcystis* control^10^,^11^,^12^. However, several species have been recorded in the *Microcystis* genus^23^ and the growth, physiology, and toxicity of *Microcystis* species varies greatly^24^. The effects of temperature, nutrients, and iron on growth of various *Microcystis* species differs significantly^25^,^26^. Moreover, *M. wesenbergii, M. flos-aquae*, and *M. viridis* have been reported as the dominant species in lakes besides *M. aeruginosa*^27^,^28^. Succession is always observed in these species in lakes^29^. A serious and important question is whether or not we can use the Cu^2+^ dose determined from *M. aeruginosa* to control all *Microcystis* species in lakes.

Even though species-dependent variation in algal sensitivity to chemical compounds has been widely reported^30^,^31^, significant differences could not be inferred because the species used in the previous study came from a different genus; however, we are talking about species in the same genus. The aim of this study was to evaluate interspecific differences in the responses of various *Microcystis* species to varying Cu^2+^ concentrations. The efficiency of primary conversion of light energy of PS II (Fv:Fm), cell viability (analyzed by 2,3,5-triphenyltetrazolium chloride (TTC) reduction), superoxide dismutase (SOD), and malonaldehyde (MDA) were analyzed to assess the physiological status of various *Microcystis* species to varying Cu^2+^ concentrations since the mechanisms by which Cu^2+^ inhibits growth of *Microcystis* had been well studied^18^,^19^,^21^.

## Materials and Methods

### Organisms

Four *Microcystis* species were provided by the Freshwater Algae Culture Collection of the Institute of Hydrobiology, Chinese Academy of Sciences. The identification numbers and origins of the four species are listed in Table 1. All strains were unicellular and purified through the dilution method and axenically cultivated in BG-11 medium for more than 3 months.

### Experimental design

Each strain was batch-cultured axenically in triplicate in 150 mL sterilized liquid BG-11 medium in a 250-mL conical flask under a 12:12-h light:dark cycle. All cultures were prepared in triplicate. The medium was treated with varying amounts of copper sulfate and the Cu^2+^ concentrations were 0.05, 0.10, 0.25, and 0.50 mg L^−1^. The culture without copper sulfate treatment was used as the control. The initial cell density of *Microcystis* was 100 × 10^4^ cells mL^−1^. The light intensity was 50 μmol photons m^−2^s^−1^ and the culture time was 4 days. The cell density and efficiency of primary conversion of light energy of PS II (Fv:Fm) was analyzed daily. At the end of the experiment, cell viability (analyzed by 2,3,5-triphenyltetrazolium chloride (TTC) reduction), superoxide dismutase (SOD), and malonaldehyde (MDA) were analyzed.

### Cell counting

The cells were counted at least three times in a hemocytometer at 400× magnification with an optical microscope (Olympus CX31; Olympus Corporation, Japan). Counting was stopped when three counts that differed by less than 10% had been obtained. The final cell density was calculated from the average of the three counts.

### Biochemical analysis

A 10-mL sample was injected into a 10-mL centrifugal tube. All of the tubes were left undisturbed in the dark at room temperature for 15 min. The sample was then analyzed by AquaPen-P 100 (Photon Systems Instruments, Czech Republic) to determine the Fv:Fm value. TTC, SOD, and MDA were analyzed according to the methods described by Hong *et al*.^32^, Choo *et al*.^33^, and Kong *et al*.^34^, respectively.

### Data analysis

All of the data are presented as mean ± SD. The specific growth rate was calculated by equation (1):


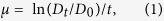


where *D*_*t*_ is the cell density at time *t, D*_0_ is the cell density in the initial logarithmic growth phase, and *t* is the duration of the logarithmic growth phase. In the current study, the value of *t* was 4.

The half maximal effective concentration (EC_50_) was determined on day 4 at the 50% inhibition rate according to the relationship between inhibition rate and concentration of Cu^2+^. The inhibition rate was calculated by equation (2):


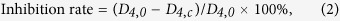


where *D*_*4,0*_ is the cell density in the control on day 4, *D*_*4,c*_ is the cell density on day 4 treated with concentration *c* of Cu^2+^.

## Results

### *Microcystis* growth

**Figure 1:**
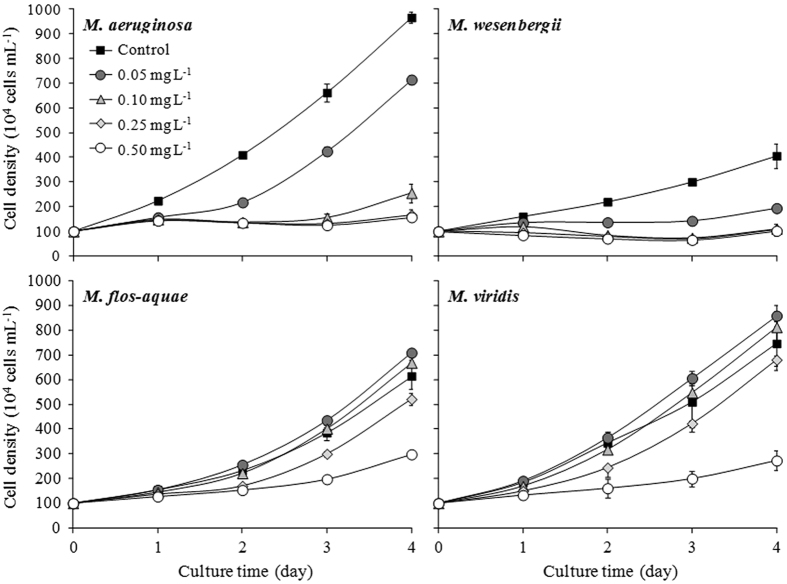
Growth curves of four *Microcystis* species in different treatments with varying Cu^2+^ concentrations.

*Microcystis* cell densities in the control increased with time and the maximum cell density of four species was in the order of *M. aeruginosa* > *M. viridis* > *M. flos-aquae* > *M. wesenbergii* (Fig. 1). The growth of these four *Microcystis* species was inhibited when treated with 0.50 mg L^−1^ Cu^2+^. It was also found that 0.05 mg L^−1^ and 0.10 mg L^−1^ Cu^2+^ promoted *M. flos-aquae* and *M. viridis* growth.

The specific growth rates were 0.57, 0.35, 0.45, and 0.50 day^−1^ in the control for *M. aeruginosa, M. wesenbergii, M. flos-aquae*, and *M. viridis*, respectively (Table 2). The specific growth rates of *M. aeruginosa* and *M. wesenbergii* decreased with increasing Cu^2+^ concentration. However, the specific growth rates of *M. flos-aquae* and *M. viridis* increased when treated with 0.05 mg L^−1^ and 0.10 mg L^−1^ Cu^2+^. Their growth rate was 0.27 and 0.25 day^−1^ in the treatment with the highest level of Cu^2+^. The EC_50_ values for *M. aeruginosa, M. wesenbergii, M. flos-aquae*, and *M. viridis* were 0.16, 0.09, 0.49, and 0.45 mg L^−1^ Cu^2+^, respectively.

### Photosynthetic activity of *Microcystis*

**Figure 2:**
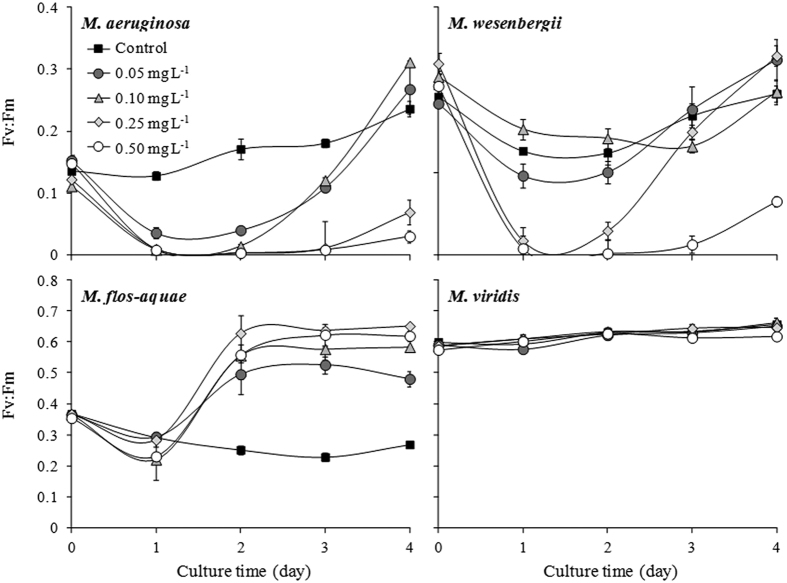
Variations in Fv:Fm in four *Microcystis* species in different treatments with varying Cu^2+^ concentrations.

The initial Fv:Fm values for *M. aeruginosa, M. wesenbergii, M. flos-aquae*, and *M. viridis* were 0.14, 0.26, 0.36, and 0.58, respectively. The Fv:Fm values decreased in all of the treatments on the first day and then increased, except for *M. viridis* (Fig. 2). There was no change in the *M. viridis* Fv:Fm value throughout the experiment. At the end of the experiment, Fv:Fm of both *M. aeruginosa* and *M. wesenbergii* decreased in the 0.50-mg L^−1^ Cu^2+^ treatment. The *M. flos-aquae* Fv:Fm value increased one-fold on day 4 in the 0.50-mg L^−1^ Cu^2+^ treatment compared with the control.

### Relative *Microcystis* TTC reduction

**Figure 3:**
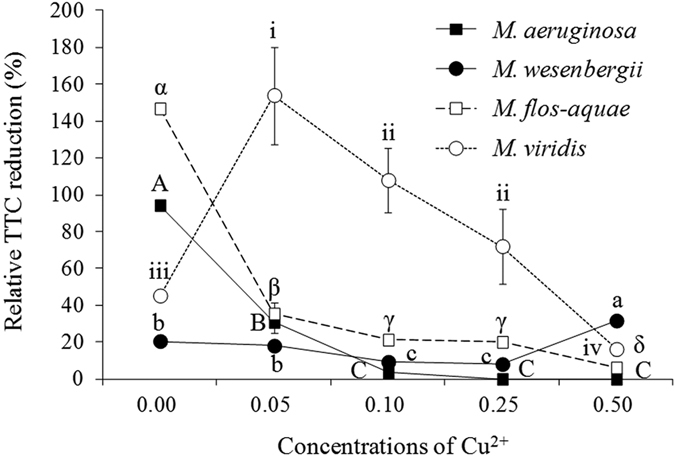
Relative TTC reduction in four *Microcystis* species treated with varying Cu^2+^ concentrations for 4 days.

Relative TTC reduction in *M. aeruginosa* and *M. flos-aquae* significantly (*P* < 0.05) decreased along with increasing Cu^2+^ concentration (Fig. 3). However, only very small changes were observed in *M. wesenbergii*. In the 0.05-mg L^−1^ Cu^2+^ treatment, the relative TTC reduction in *M. viridis* reached 160% compared with 40% in control.

### *Microcystis* SOD and MDA content

**Figure 4:**
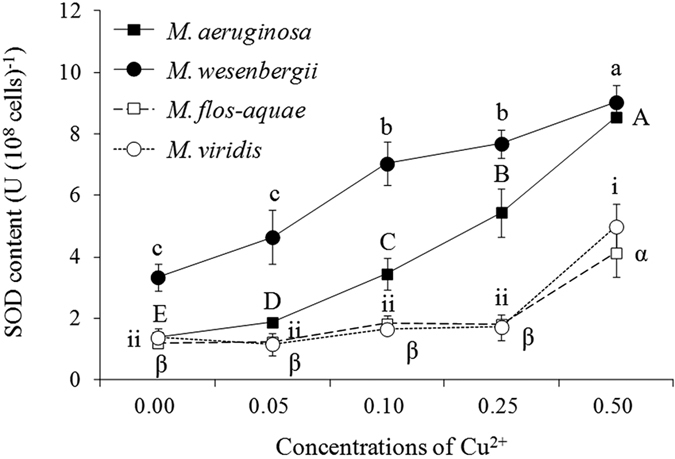
SOD content in four *Microcystis* species treated with varying Cu^2+^ concentrations for 4 days.

**Figure 5:**
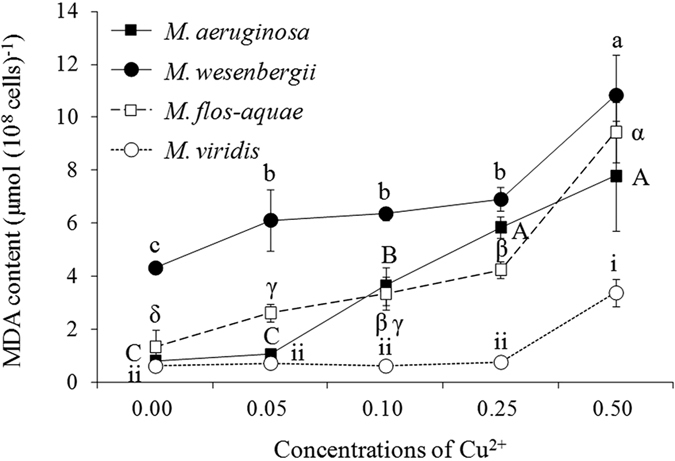
MDA content in four *Microcystis* species treated with varying Cu^2+^ concentrations for 4 days.

In the control, SOD content in *M. wesenbergii* was greater than that in the other species (Fig. 4). With increasing Cu^2+^ concentration, SOD content significantly increased in all four species (*P* < 0.05). In the highest Cu^2+^ treatment, SOD content was 9.0, 8.5, 5.0, and 4.1 U (10^8^ cells)^−1^, for *M. wesenbergii, M. aeruginosa, M. flos-aquae*, and *M. viridis*, respectively. MDA content also significantly increased with increasing Cu^2+^ concentration in all four species (*P* < 0.05; Fig. 5). In the 0.50-mg L^−1^ Cu^2+^ treatment, MDA content was 10.8, 9.4, 7.8, and 3.4 μmol (10^8^ cells)^−1^ for *M. wesenbergii, M. flos-aquae, M. aeruginosa*, and *M. viridis*, respectively.

## Discussion

Our results reveal species-dependent variation in the sensitivity of *Microcystis* species to copper sulfate. The species-dependent variation in algal sensitivity to copper was also recently reported by Tsai^35^. The 96-h-EC_50_ value in *Microcystis* species to Cu^2+^ was in the order of *M. flos-aquae* > *M. viridis* > *M. aeruginosa > M. wesenbergii* and ranged from 0.09 to 0.49 mg L^−1^. The decrease in Fv:Fm ratio in the highest Cu^2+^ treatment in the current study was *M. flos-aquae < M. viridis* < *M. aeruginosa < M. wesenbergii* (Fig. 2), which was consistent with the order of EC_50_.

It can be seen that Fv:Fm ratio of *M. aeruginosa* decreased with increase of Cu^2+^ concentration but Fv:Fm ratio of *M. flos-aquae* and *M. Wesenbergii* decreased in first 2 days of application of copper sulfate, and then increased as the days progress. For *M. viridis*, the ratio was at par with the control in all the treatments. These differences would be because of variations in sensitivity to Cu^2+^ among various species. Although, it was reported that high level Cu^2+^ reduced the electron transfer rate of the PS II system in *M. aeruginosa*^22^,^36^, inhibition of PS II system may not be the only way by which copper sulfate controls other *Microcystis* species. The decrease in the relative TTC reduction reflected cell damage exposed to Cu^2+^. Our results showed that cells of all the *Microcystis* species were damaged when exposed to Cu^2+^ except for *M. viridis* (Fig. 3). This result was consistent with the results of growth and Fv:Fm ratio.

SOD may be crucial to the growth inhibition of *Microcystis*^37^,^38^. *M. wesenbergii* had the highest SOD content in the control compared with the other *Microcystis* species (Fig. 4). However, the *M. wesenbergii* EC_50_ value was the lowest. Moreover, *M. aeruginosa, M. flos-aquae*, and *M. viridis* EC_50_ values varied greatly but their SOD contents were similar. The MDA content was considered an indicator of cell injury and increasing MDA indicated damage of cytomembrane^39^. In the current study, the values did not decease with increasing SOD content induced by increasing Cu^2+^. All of the above results suggest that SOD failed to reduce Cu^2+^ damage in *Microcystis* in the current study. Both enzymatic and non-enzymatic antioxidants of *Microcystis* played important roles in tolerating oxidative damage^37^,^38^. Therefore, non-enzymatic antioxidants such as reduced glutathione (GSH) and ascorbic acid (AsA) would be important for *Microcystis* spp. to counteract the oxidative stress induced by Cu^2+ ^38.

The initial *M. wesenbergii* SOD and MDA content was significantly higher than that of the other species. Temperature may have affected this, given that the optimal temperature for *M. wesenbergii* growth is approximately 30 °C^25^ and the temperature in our experiment was much lower (25 °C). Both SOD and MDA content increased with increasing concentrations of Cu^2+^ in the current study. Similar result was also reported by Chen *et al*.^39^ and Shao *et al*.^40^. However, it was considered that SOD and MDA had an rough inverse relationship^38^. It might be because that MDA was a continuously accumulating material but SOD varied against time^41^. As shown in Fig. 2, damage from Cu^2+^ was highest on first day and then the physiological activity was improving later. Therefore, the relationship between SOD and MDA on day 4 was irregular.

Extracellular polysaccharide (EPS) release is another protective response against chemical compounds in algae including microcystins^42^, salt^43^, and heavy metals^44^,^45^,^46^. Li *et al*.^47^ suggested that EPS is an important strategy to reduce Cu^2+^ damage because –COO^−^ and some amino groups in EPS can absorb heavy metals effectively^48^. Xu *et al*.^49^ demonstrated that EPS content was significantly lower in *M. wesenbergii* than the other three *Microcystis* species under standard culture conditions similar to ours. It could be deduced from their results that *M. wesenbergii* was the most sensitive species. This conclusion is also supported by our results (Table 2). Therefore, the species-dependent variation in the sensitivity of *Microcystis* species to copper sulfate in the current study may have been the result of variations in EPS content in different *Microcystis* species. Forni *et al*.^50^ reported that the content of polysaccharide of *M. viridis* was much higher than other *Microcystis* species. This difference would cause variation in growth and physiology of *Microcystis* when treated with copper sulfate. It was noticed that the standard deviation of cell density obtained with *M. viridis* was much higher than other species and this result supported above deduction. In addition, it was also found that the standard deviation of TTC, SOD and MDA obtained with *M. viridis* was very high. Polysaccharide may be the main interfering substance for analysis of above enzyme activity. However, the error range was still within the equivalent range reported by some other researchers^37^,^39^,^40^.

The growth curves of *M. flos-aquae* and *M. viridis* exposed to 0.05 and 0.10 mg L^−1^ Cu^2+^ were higher even than control (Fig. 1). This was due to that Cu^2+^ are essential micronutrient for *Microcystis*^51^. Additionally, it has also been well documented that low-level contaminants promote *Microcystis* growth^52^,^53^,^54^. However, the sensitivity or tolerance to heavy metals varies amongst different algae and this variation caused that the beneficial concentration of Cu^2+^ for *M. flos-aquae* and *M. viridis* inhibited growth of *M. aeruginosa* and *M. wesenbergii*. It was also noticed that 0.10 mg L^−1^ Cu^2+^, which promoted growth of *M. flos-aquae* and *M. viridis*, was higher than the *M. wesenbergii* EC_50_. This indicated that Cu^2+^ doses that control *M. wesenbergii* promote *M. flos-aquae* and *M. viridis* growth in lakes. In Fairmont Lakes (USA), 0.033–0.054 mg L^−1^ Cu^2+^ was used for over 58 years to control algal growth^14^. The recommended dose of Cu^2+^ to control cyanobacterial blooms in Canada was 0.05–0.125 mg L^−1^ 55. These Cu^2+^ concentrations would stimulate *M. flos-aquae* and *M. viridis* growth. Therefore, the actual amount of Cu^2+^ used to control *M. wesenbergii* should be much greater than 0.10 mg L^−1^; while the correct dose for *M. flos-aquae* and *M. viridis* control requires investigation in lakes. In addition, the species of copper from different copper algaecides should also be considered^56^.

The risk of microcystin release when *Microcystis* membranes are destroyed by Cu^2+^ treatment is an important concern in the application of copper sulfate to control *Microcystis*. Trace Cu^2+^ (0.16–0.50 mg L^−1^)^10^,^11^,^21^ can result in cell lysis and microcystin release. The most sensitive species, *M. wesenbergii*, potentially produces microcystins and other toxins^57^,^58^. This species always dominates in summer in lakes with high biomass^27^. Nevertheless, it has been reported as a non-microcystin production species in China and other countries^59^,^60^. Therefore, 0.10–0.16 mg L^−1^ Cu^2+^ would reduce *M. wesenbergii* growth without cell lysis and microcystin release. Additionally, this Cu^2 + ^dose would not promote growth in other *Microcystis* species.

*M. aeruginosa* and *M. viridis* colonies may produce large amounts of microcystins^58^,^61^. The EC_50_ values of these two species to Cu^2+^ in this study were 0.16 and 0.45 mg L^−1^, respectively. These concentrations may induce cell lysis and microcystin release. Reports of toxin production are rare in *M. flos-aquae* (sometimes identified as *M. ichthyoblabe*)^25^,^57^. This species always dominates in lakes before early summer and the biomass is lower compared with *M. wesenbergii* in midsummer^62^. *M. flos-aquae* growth would be inhibited by 1 mg L^−1^ Cu^2+^, which is safe for drinking water and there is no risk of microcystin release. Therefore, copper sulfate treatment for *Microcystis* control could be applied before midsummer when *M. aeruginosa* and *M. viridis* are not the dominant species. The dose of copper sulfate should be evaluated according to the dominant *Microcystis* species.

The EC_50_ value is also affected by initial cell density^63^,^64^ and *Microcystis* phenotype^36^,^47^. In the current study, all of the strains were unicellular and the initial cell density was the same. The effects of initial cell density and *Microcystis* phenotype on EC_50_ were excluded. However, the EC_50_ values obtained in the current study should be re-evaluated because *Microcystis* always exists as colonies in lakes with varying cell densities^65^. The effects of temperature on heavy metal tolerance in *Microcystis* should also be considered^66^.

## Conclusions

Our results reveal species-dependent variation in the sensitivity of *Microcystis* species to copper sulfate. The 96-h-EC_50_ value of *Microcystis* species to Cu^2+^ was in the order of *M. flos-aquae* > *M. viridis* > *M. aeruginosa > M. wesenbergii* and ranged from 0.09 to 0.49 mg L^−1^. MDA content did not decrease with increasing SOD content induced by increasing Cu^2+^, suggesting that SOD failed to reduce Cu^2+^ damage to *Microcystis* in the current study. However, the species-dependent variation in the sensitivity of *Microcystis* species to copper sulfate may have resulted from variations in EPS content in different *Microcystis* species. *M. flos-aquae* and *M. viridis* growth were promoted when exposed to 0.05 and 0.10 mg L^−1^ Cu^2+^. Our results suggest that copper sulfate treatment for *Microcystis* control could be applied before midsummer when *M. aeruginosa* and *M. viridis* are not the dominant species and the actual amount of Cu^2+^ used to control *M. wesenbergii* should be much greater than 0.10 mg L^−1^, while *M. flos-aquae* and *M. viridis* control in lakes requires further investigation.

## Additional Information

**How to cite this article:** Wu, H. *et al*. Species-dependent variation in sensitivity of *Microcystis* species to copper sulfate: implication in algal toxicity of copper and controls of blooms. *Sci. Rep.* **7**, 40393; doi: 10.1038/srep40393 (2017).

**Publisher's note:** Springer Nature remains neutral with regard to jurisdictional claims in published maps and institutional affiliations.

## Figures and Tables

**Table 1:** Details of the four *Microcystis* species used in the current study.

Species	Code	Culture collection	Origin	Morphology
*M. aeruginosa*	1343	FACHB	Lake Taihu, China	Unicellular
*M. wesenbergii*	1324	FACHB	Lake Taihu, China	Unicellular
*M. flos-aquae*	1272	FACHB	Lake Taihu, China	Unicellular
*M. viridis*	1337	FACHB	Lake Dianchi, China	Unicellular

**Table 2:** Specific growth rate (day^−1^) and EC_50_ (mg L^−1^) in four *Microcystis* species exposed to varying Cu^2+^ concentrations.

	Specific growth rate in treatments with various concentrations of Cu^2+^ (mg L^−1^)					EC_50_
	Control	0.05	0.10	0.25	0.50	
*M. aeruginosa*	0.57	0.49	0.23	0.13	0.11	0.16
*M. wesenbergii*	0.35	0.17	0.03	0.02	0.00	0.09
*M. flos-aquae*	0.45	0.49	0.47	0.41	0.27	0.49
*M. viridis*	0.50	0.54	0.52	0.48	0.25	0.45
